# Supplementary material for: Prognosis Associated with Sub-Types of Hyperglycaemia in Pregnancy
Source: J Clin Med. 2021 Aug 30;10(17):3904. doi: 10.3390/jcm10173904 (PMC8432067; doi:10.3390/jcm10173904)
Supplement: Supplementary file 1 [file jcm-10-03904-s001.zip › Figure S1 Flow chart.pptx]

## Slide 1
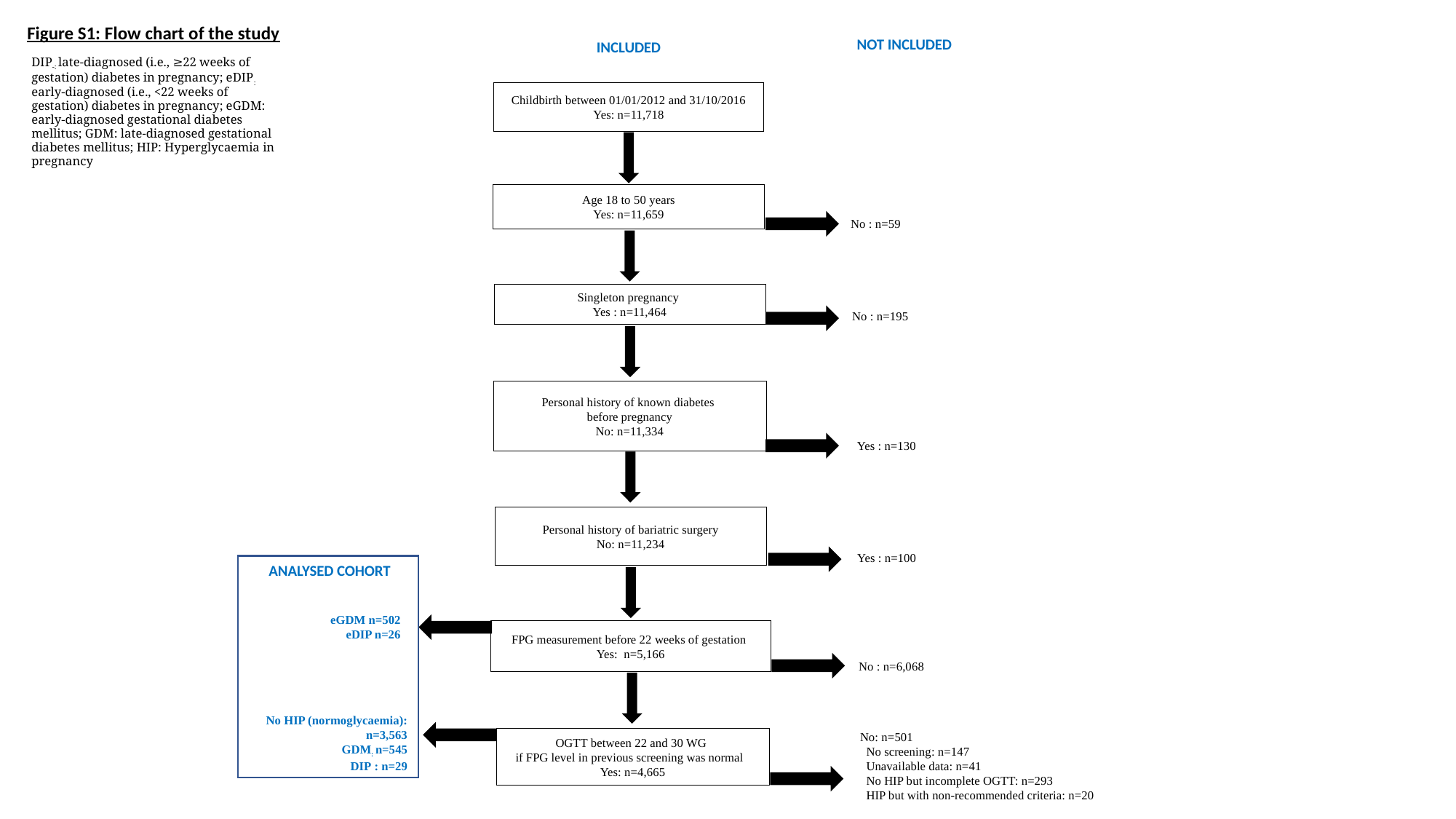

Figure S1: Flow chart of the study
NOT INCLUDED
INCLUDED
#
DIP-: late-diagnosed (i.e., ≥22 weeks of gestation) diabetes in pregnancy; eDIP: early-diagnosed (i.e., <22 weeks of gestation) diabetes in pregnancy; eGDM: early-diagnosed gestational diabetes mellitus; GDM: late-diagnosed gestational diabetes mellitus; HIP: Hyperglycaemia in pregnancy
 Childbirth between 01/01/2012 and 31/10/2016
Yes: n=11,718
Age 18 to 50 years
Yes: n=11,659
No : n=59
Singleton pregnancy
Yes : n=11,464
No : n=195
Personal history of known diabetes
before pregnancy
No: n=11,334
Yes : n=130
Personal history of bariatric surgery
No: n=11,234
Yes : n=100
ANALYSED COHORT
eGDM n=502
eDIP n=26
FPG measurement before 22 weeks of gestation
Yes: n=5,166
No : n=6,068
No HIP (normoglycaemia): n=3,563
GDM: n=545
DIP : n=29
No: n=501
 No screening: n=147
 Unavailable data: n=41
 No HIP but incomplete OGTT: n=293
 HIP but with non-recommended criteria: n=20
OGTT between 22 and 30 WG
if FPG level in previous screening was normal   Yes: n=4,665
Figure 1 : Flowchart de la sélection de la population de l’étude
