# Supplementary material for: Prognosis Associated with Sub-Types of Hyperglycaemia in Pregnancy
Source: J Clin Med. 2021 Aug 30;10(17):3904. doi: 10.3390/jcm10173904 (PMC8432067; doi:10.3390/jcm10173904)
Supplement: Supplementary file 1 [file jcm-10-03904-s001.zip › Table S1.pdf]

Table S1: Characteristics of women included and those not included in the study.

	Included	Not included	p-value
	n=4,665	n=6,569	
**Screening for HIP before 22 WG**			
Fasting plasma glucose (mmol/L)	4.6 (0.6)	4.7 (0.6)	<0.0001
Gestational age at HIP screening (WG)	12.2 (4.2)	13.0 (5.3)	<0.0001
**Screening with OGTT at 22 WG or later**			
Fasting plasma glucose (mmol/L)	4.4 (0.5)	4.5 (0.6)	<0.0001
1-hour plasma glucose (mmol/L)	7.0 (1.8)	7.1 (1.9)	0.0067
2-hour plasma glucose (mmol/L)	6.1 (1.5)	6.2 (1.6)	0.3672
Gestational age at OGTT (WG)	27.2 (3.0)	28.0 (5.6)	<0.0001
**Metabolic characteristics**			
Age (years)	30.7 (5.5)	29.9 (5.6)	<0.0001
Pre-pregnancy body mass index (kg/m2)	25.2 (5.1)	24.8 (5.0)	<0.0001
Family history of diabetes	1,291 (27.7)	1,631 (24.8)	0.0007
Employment at beginning of pregnancy	1,887 (40.5)	2,436 (37.2)	0.0003
Parity	2.1 (1.2)	2.2 (1.3)	0.1158
**Previous pregnancy (ies)**			
History of hyperglycaemia in pregnancy			<0.0001
First child	1,814 (38.9)	2,449 (37.3)	
No	2,561 (54.9)	3,851 (58.6)	
Yes	290 (6.2)	269 (4.1)	
History of large-for-gestational-age infant			0.0016
First child	1,814 (38.9)	2,449 (37.3)	
No	2,688 (57.6)	3,957 (60.2)	
Yes	163 (3.5)	163 (2.5)	
History of hypertensive disorders			0.0369
First pregnancy	1,265 (27.1)	1,686 (25.7)	
No	3,281 (70.3)	4,754 (72.4)	
Yes	119 (2.6)	129 (2.0)	
History of foetal death			0.0192
First pregnancy	1,265 (27.1)	1,686 (25.7)	
No	3307 (70.9)	4707 (71.7)	
Yes	93 (2.0)	176 (2.7)	
Ethnicity			<0.0001
European	1,269 (27.3)	1,874 (28.6)	
African	881 (18.9)	1,325 (20.2)	
North African	1,378 (29.6)	1,885 (28.7)	
Asian	96 (2.1)	165 (2.5)	
Caribbean	270 (5.8)	369 (5.6)	
Indian-Pakistan-Sri Lankan	519 (11.2)	531 (8.1)	
Other	243 (5.2)	410 (6.3)	

Data are n (%) or mean (standard deviation)
